# Dystrophin genetic variants and autism

**DOI:** 10.1007/s44192-022-00008-z

**Published:** 2022-03-24

**Authors:** Maria Rita Passos-Bueno, Claudia Ismania Samogy Costa, Mayana Zatz

**Affiliations:** Departamento de Genética e Biologia Evolutiva, Centro de Estudos do Genoma Humano e Células-Tronco, Instituto de Biociências, Universidade de São Paulo, São Paulo, SP Brazil

## Abstract

Loss-of-function variants in the dystrophin gene, a well-known cause of muscular dystrophies, have emerged as a mutational risk mechanism for autism spectrum disorder (ASD), which in turn is a highly prevalent (~ 1%) genetically heterogeneous neurodevelopmental disorder. Although the association of intellectual disability with the dystrophinopathies Duchenne (DMD) and Becker muscular dystrophy (BMD) has been long established, their association with ASD is more recent, and the dystrophin genotype-ASD phenotype correlation is unclear. We therefore present a review of the literature focused on the ASD prevalence among dystrophinopathies, the relevance of the dystrophin isoforms, and most particularly the relevance of the genetic background to the etiology of ASD in these patients. Four families with ASD-DMD/BMD patients are also reported here for the first time. These include a single ASD individual, ASD-discordant and ASD-concordant monozygotic twins, and non-identical ASD triplets. Notably, two unrelated individuals, which were first ascertained because of the ASD phenotype at ages 15 and 5 years respectively, present rare dystrophin variants still poorly characterized, suggesting that some dystrophin variants may compromise the brain more prominently. Whole exome sequencing in these ASD-DMD/BMD individuals together with the literature suggest, although based on preliminary data, a complex and heterogeneous genetic architecture underlying ASD in dystrophinopathies, that include rare variants of large and medium effect. The need for the establishment of a consortia for genomic investigation of ASD-DMD/BMD patients, which may shed light on the genetic architecture of ASD, is discussed.

## Introduction

Autism spectrum disorder (ASD), or autism, is a neurodevelopmental disorder characterized by impairment of social communication and interaction, sensory anomalies and repetitive behaviors. In addition to these core symptoms, other neurological or psychiatric conditions, such as varying levels of intellectual disability, epilepsy, attention-deficit/hyperactivity (ADHD), anxiety and obsessive–compulsive disorders (OCD), often co-occur with ASD [1]. Data based mainly on the population of European ancestry estimate a prevalence of about 1%, being four times more frequent in males [2, 3].

Twin, families and population studies have shown that ASD is highly heritable [4–8]. In the past decades, large-scale genomic investigation in ASD individuals has contributed to a better understanding of the genetic architecture of ASD [9, 10]. The most recent ASD genome wide association studies (GWAS) with large data sets have identified few associated SNPs with high confidence, but there is still a lot to be dissected on the ASD genetic architecture under oligogenic or polygenic models [10]. Notably, the investigation on de novo rare variants (DNVs) in probands through trio analysis (mother, father and proband) has been the most successful approach to uncover ASD genes [11–13]. This approach has revealed an enrichment of both single nucleotide variants (SNVs) and copy number variation (CNVs) in ASD individuals compared to their unaffected sibs [14, 15]. Interestingly, the known and candidate ASD genes converge on a few molecular pathways, such as cell adhesion, neuronal orientation and growth, neurotransmission, cell signaling and transcription, protein translation and degradation, as well as chromatin remodeling and cytoskeleton. These functions may ultimately affect the neuronal plasticity, functioning of synaptic networks and neuronal connectivity in the brain [4, 11, 14, 16].

Most of the rare variants with large clinical effects are identified in syndromic ASD forms with intellectual disability (ID) and other comorbidities, and are associated with Mendelian forms of ASD in 10–30% of patients [4, 17]. There is a large overlap between ASD and ID genes, but some are more likely to cause ASD, such as *CHD8* and *KMTC2.* Moreover, the variants in genes associated with syndromic forms of ASD (such as *SYNGAP1* among others) are rare, with a prevalence of less than 0.1% [10, 11]. Exceptions to this include Phelan-McDermid Syndrome (PMS, #MIM 606232), first characterized in the 1990’s [18] and one of the most prevalent forms of autism and/or ID, with a prevalence varying from 0.5% to 2% in Brazilian, European and USA ASD/ID cohorts [19, 20].

In addition to DNVs associated with monogenic conditions, rare inherited pathogenic risk variants also contribute to the etiology of ASD [15, 21, 22], as exemplified by SNVs or CNVs, respectively at *SHANK2* and at 16p12.1. Such variants are usually associated with incomplete penetrance and great clinical variability, and they are believed to increase the risk of manifesting ASD by sensitizing the nervous system. Additionally, the manifestation of ASD may depend on the accumulation of other risk factors, including genetic variants of medium or lower effect (see Ref. [23] for a discussion about ASD modifiers) [24–31]. Interestingly, pathogenic variants in the dystrophin (*DMD)* gene, responsible for Duchenne muscular dystrophy (DMD) and its allelic and less severe form Becker muscular dystrophy (BMD) have been recognized as risk variants for ASD. In 2010, one of the seminal papers employing genome-wide CNV scan revealed three individuals with CNVs in the dystrophin gene in a cohort of 996 ASD families [30], thus in a much higher frequency than the DMD prevalence of 1:3.500–5000 males [32].

The first and most studied neurodevelopmental disorder in DMD/BMD individuals has been ID [33–41]. The relationship between DMD and cognitive impairment has been recognized since the early descriptions of the disease [42, 43]. Nowadays it is widely recognized that ID affects around 30% of individuals with DMD, while at a lower frequency in BMD (up to 12%), but higher than in the general population [44–48]. More recently, systematic studies with DMD cohorts have also shown a higher frequency of other neurodevelopmental disorders (NDDs) and neuropsychological conditions, such as ASD (up to 21%, [49]), ADHD (~ 30%, [50]), OCD (~ 5%, [50]) and epilepsy (up to 6%, [51]), among others [52]. Considering the high frequency of ASD within dystrophinopathies, together with the still incipient data on this topic, particularly on the potential relevance of the genetic background to its etiology, this review will focus on updating the ASD prevalence among DMD/BMD cohorts, dystrophin in the brain and genotype–phenotype correlations between dystrophin isoforms, genetic background and ASD in dystrophinopathies. We also describe six ASD individuals with dystrophin loss-of-function (LoF) variants from four unrelated families identified in our center, in whom we also explored the contribution of the genetic background to ASD. Notably, two of them were primarily ascertained because of the ASD phenotype, with no complaint of muscle weakness at the original evaluation.

## Dystrophinopathies: general aspects

DMD and BMD are allelic conditions distinguished by the age of onset of clinical manifestations, rate of progression and life expectancy. The most severe form of DMD is the commonest form, affecting about 1 in 3500–5000 boys, while BMD is rarer, affecting 1 in ~ 20,000 males [53, 54].

DMD and BMD are caused by LoF variants in dystrophin, the largest gene of the human genome, containing 79 exons and over 2.5 million bp of genomic sequence. It encodes the dystrophin protein [35]. Deletions and duplications correspond to about 60–80% of the pathogenic variants, while the remaining ones include nonsense, splice site and intronic variants [55–59]. The type of variant and severity of the disease are strongly correlated: usually, variants creating premature stop codons are associated with the more severe DMD, while in-frame deletions are more likely associated with the milder BMD, however, depending also on the variant site within the dystrophin gene [35, 56, 58, 59].

**Fig. 1:**
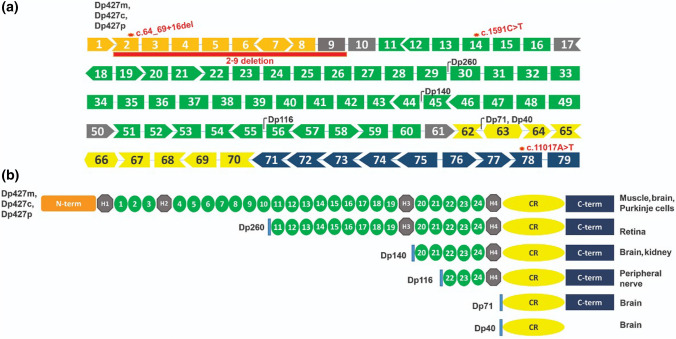
Schematic representation of DMD exons gene and main protein isoforms. **a** Representation of the 79 exons of the dystrophin gene. Exons’ format represents whether splicing between adjacent exons maintain the protein’s ORF (open read frame) or create out of frame, truncated, proteins. A black arrow indicates the initiation of the main isoforms. The longest isoforms, Dp427m, Dp427p and Dp427c, differ only for their first exon and are named according to their principal site of expression. In addition, four internal promoters, found in different introns of the larger isoforms, control the expression of the smaller isoforms Dp260, Dp140, Dp116, and Dp71/Dp40, named after their molecular weight. DMD variants here described are represented (written in red): three SNVs affecting exons 2 (family 3), 14 (family 4), 78 (family 2) and a 2–9 exons deletion (family 1). Exons’ colors represent the full-length isoform main domains of the dystrophin protein (which is shown in **b** orange = N-terminus (which contains the actin-binding domain); grey = hinge domains; green = ROD domain, composed of 24 spectrin-like domains; yellow = the cysteine rich domain; and dark blue = C-terminus portion. **b** Representation of the dystrophin isoforms with their domains and the main tissue that they are expressed in. In addition to the ones represented, alternative splicing patterns and polyadenylation sites create a greater number of dystrophin isoforms, increasing its complexity. The smallest isoform, Dp40, shares the Dp71 promoter region, but differs by its truncated 3′ portion

Dystrophin is mainly expressed in skeletal and cardiac muscles, but also in the central nervous system (CNS). Three of its seven independent promoters are located at the 5′ end of the gene and regulate the expression of long, full length 14 kb transcripts mainly in muscle, brain and Purkinje cells (Dp427m, Dp427c, and Dp427p, respectively), and differing between each other only by the first exon [35]; the other four promoters are located along the dystrophin gene and regulate the transcription of smaller isoforms (Dp260, Dp140, Dp116 and Dp71), which are expressed in muscle and brain or only in the brain (summarized in Fig. 1). These smaller isoforms can also present differential polyadenylation sites and alternative splicing patterns in addition to a unique first exon, considerably increasing their complexity, particularly in the CNS. Finally, the isoform Dp40 shares the same promoter with Dp71, but presents a differential splicing pattern, lacking the C-terminal of Dp71 [60, 61]. The N-terminal of the long forms and some of the Dp71 dystrophin transcripts interact with the sarcomeric network by binding to F-actin [62, 63]. The C-terminal of all isoforms is associated with the plasma membrane through a complex of glycoproteins (The Dystrophin Glycoprotein Complex, DGC). In skeletal muscle, the primary role of dystrophin is mechanical stabilization of the plasma membrane, protecting the muscle fibers from long-term contraction-induced damage and necrosis [35, 56]. Together with the GC, dystrophin also plays a role in the communication with the extracellular medium, thus acting as a transmembrane signaling complex [56].

Dystrophin isoforms show a complex expression pattern in the CNS, depending on cell type, developmental stage and expression levels [64, 65]. The Dp427 isoforms are part of the DGC, however, contrary to muscle tissue, different DGC-like complexes exist in the CNS, and their proteins’ composition and interactions, as well as their function, seem to vary depending on cell type [66–70]. Over the years, Dp140 and Dp71 isoforms have gained more attention in the literature regarding neurodevelopment than the other dystrophin isoforms due studies uncovering positive genotype–phenotype correlation with ID, their high expression in the brain, and function [71, 72]. They may also be involved with neuronal differentiation, chromatin remodeling, vascular development, and synaptic and astrocyte function, which are commonly altered in ASD patients [64, 65, 73]. Finally, as reviewed by different authors, neuroimmune modulation impairment [74, 75], cerebellar dysfunction [76] and unbalance of inhibitory/excitatory components [77, 78] are suggested as potential molecular mechanisms explaining cognitive and behavioral phenotypes among DMD/BMD individuals.

## ASD prevalence in DMD/BMD

One of the first systematic studies on ASD prevalence in dystrophinopathies was published by Wu et al. [79]. They reported a prevalence of 3.8% of ASD among 158 DMD boys ascertained in the Massachusetts Muscular Dystrophy Association Neuromuscular Clinic (at Children’s Hospital Boston), which was significantly increased as compared with the general population (0.0016%). In Brazil, an ASD prevalence of 20% was estimated based on a yet unpublished study with 54 clinically diagnosed DMD boys evaluated by the Childhood Autism Rating Scale (CARS) [80]. At least 19 additional studies (Table 1) have evaluated the co-occurrence of ASD in up to 2000 males with dystrophinopathies from research centers/hospitals in different countries [34, 41, 44–50, 53, 80–89]). Even though most of these studies are based on a relatively small number of cases, most of them suggested an increased ASD prevalence in both DMD and BMD boys compared to the general population, ranging from 0 to 21% among DMDs, and 0% to 11.4% among BMDs (Table 1).

**Table 1:** ASD prevalence in DMD/BMD individuals from different cohorts

Article	Number of individuals enrolled	Age of evaluation mean, (range)^b^	Method of ASD evaluation	ASD frequency	DMD Genotype-ASD phenotype correlation: Results and groups’ division
Kumagai et al. [34]^c^	137 (94 DMD and 43 DMB)	NA	NA	DMD = 8.5% (8/94) DMB = 4.7% (2/43)	Positive association. Enrichment of ASD individuals in the group carrying 3′ variants (exonic position not specified)
Wu et al. [79]	158 (DMD)	NA	Medical records followed by evaluation by a neurologist according to the Diagnostic and Statistical Manual of Mental Disorders-IV	3.8% (6/158)	NA
Darke et al. [81]	45 (37 DMD and 8 DMB)	NA	Previous formal ASD diagnosis	DMD = 5.4% (2/37) BMD = 37.5% (3/8)^d^	NA
Young et al. [45]	24 (BMD)	14.2 yo (6–43)	Previous formal ASD diagnosis	8.3% (2/24)	NA
Hendrisen et al. [50]	351 (DMD)	11.9 yo (3–38)	Previous formal ASD diagnosis reported by caregivers	3.1% (11/351)	NA
Nakamura et al. [82]^c^	200 (dystrophinopathies)	NA	Medical records	7.5% (15/200)	Positive association. Enrichment of ASD individuals in the group carrying distal variants relative to exon 44
Donders et al. [83]	22 (DMD)	11.09 yo	Previous formal ASD diagnosis	0%	NA
Hinton et al. [84]	85 (DMD)	6–16 yo (NA)	Social Communication Questionnaire followed by The Autism Diagnostic Interview-Revised	19% (16/85)	NA
Elsenbruch et al. [85]	50 (DMD)	15.4 yo (8–23)	Previous formal diagnosis	0%	NA
Banihani et al.[41]	59 (DMD)	9.8 yo (4.2–18.9)	Diagnostic and Statistical Manual of Mental Disorders criteria + ADOS	15.3% (9/59)	No association observed
Ricotti et al. [49]	87 (DMD)	NA	Developmental, Diagnostic and Dimensional Interview – short version (3Di-sv)	21% (18/87)	No association observed
Colombo et al. [86]	47 (DMD)	10.96 yo (2–18)	Child Behavior Checklist (CBCL) or DSM-IV criteria followed by ADOS	14.9% (7/47)	No association observed
Latimer et al. [46]	192 (dystrophinopathies)	3–31 yo (NA)	Previous formal ASD diagnosis reported by caregivers	DMD = 6.7% (11/164) BMD = 3.6% (1/28)	NA
Fujino et al. [47]	56 (50 DMD and 6 DMB)	12.9 yo (NA)	Pervasive Developmental Disorders/Autism Spectrum Disorders Rating Scale (PARS)	19.6 (11/56)	No association observed
Madanelo [80]	54 (DMD)	10.9 yo (5–17)	Childhood Autism Rating Scale	20% (11/54)	No association observed
Mori-Yoshumura et al. [44]	125 (DMB)	37.2 yo (20–72)	Self-report questionnaire indicating previous formal ASD diagnosis	0.08% (1/125)	NA
Thangaraj et al. [87]	173 (DMD)	6.4 yo (4–9)	Previous formal diagnosis reported by caregivers	2.9% (5/173)	NA
Lambert et al. [48]	70 (DMB)	13.4 yo (1–36.7)	Autistic features evaluated based on clinical records	11.4% (8/70)	No association observed
Thangaraj et al. [88]	196 (DMD)	5.8 (4.1–8)	Previous formal diagnosis	1.5% (3/196)	No association observed
Thangaraj et al. [89] ^e^	27 (DMD)	NA	Previous formal ASD diagnosis reported by caregivers	0%	NA
Tesei et al. [53]	101 (84 DMD and 17 DMB)	DMB = 20.24 (NA) DMD = 16.01 (NA)	Previous formal diagnosis or screening questionnaires followed by ADOS (Diagnostic Observation Schedule)	DMD = 8.3% (7/84) DMB = 0%	NA

Numbers of individuals enrolled on each study, muscle diagnosis, method of ASD assessment and its frequency, in addition to genotype–phenotype analysis are sowed

Selected articles^a^ are displayed in crescent year of publication

*NA* not available

^a^The search was performed in September/2021 in the PubMed database, using all combinations of the following terms: “autism”, “autistic” and “ASD” combined with “DMD”, “BMD”, and “Duchenne”, “Becker muscular”, “dystrophinopathies” and “dystrophinopathy”. This first analysis resulted in 51 unique entries. Next, filters were based on inclusion of studies with clinical evaluation for ASD or autistic features. Reviews were not included. Selected articles were evaluated for study type (prospective or retrospective); muscle diagnosis (DMD, BMD or dystrophinopathy, when not specified); mean age of cohort/groups' evaluation, when available; method for ASD evaluation and ASD frequency reported in the study; and genotype–phenotype analysis, in addition to its methodological approach and results, when performed

^b^*yo* years old

^c^methodological and cohort details not available

^d^This article presented the higher ASD frequency among DMBs, but due to its small sample this data was not considered in the main text

^e^The female girl of this study was not included here

Different methodological approaches to assess behavioral and psychosocial function have been applied for diagnosing ASD frequency among dystrophinopathies [47, 79, 86]. Indeed, a recent systematic review pointed out that 61 different instruments were used in 54 studies [90]. The studies included in the present review include previous/clinically recorded formal diagnosis, structured questionnaires, clinical evaluation by a specialist (psychologist, neurologist) or a combination of them, based on the current DSM at the time of evaluation (Table 1). Of note, we observed a higher ASD frequency (3.8–21%) based on cohorts prospectively evaluated using a formal ASD evaluation, such as ADOS (Autism Diagnostic Observation Schedule) or ADI-R (autism diagnostic interview-revised) than those based on medical records (0–11%) (Table 1). These results also may be related to the increased awareness of ASD in the general population in the last decades [91]. On the other hand, ASD frequency seemed to be lower in BMD patients, even among studies that evaluated both DMD and BMD [34, 46, 53], suggesting that these results are not due to methodological issues. Overall, both the literature and our observations suggest the need to standardize methodologies to bring forward more accurate ASD diagnosis and prevalence estimates.

Another interesting finding shown by different authors is that the majority of DMD patients present a mild form of ASD/ID [83, 84]. In agreement with these results, Banihani et al. [41] showed that, even though 30% of their cohort (59 DMD individuals) presented a FSIQ (Full Scale Intelligence Quotient) below 70, only 3% of the individuals were diagnosed with severe ID (FSIQ less than 50), further indicating that the NDD presentation in DMD is generally mild.

It is worth noting that ASD and cognitive/behavioral impairments have been also described in symptomatic female DMD carriers by different authors [92–95]. More recently, Demirci et al. [96] reported that carrier mothers of DMD individuals performed poorly in cognitive tests, when compared to non-carrier DMD mothers and controls. Despite the small sample (31 carriers and 24 non-carriers), this study sheds light on the hypothesis that pathogenic variants in the dystrophin gene may be associated with cognitive impairment in asymptomatic females at some level. Systematic studies are necessary to clarify the effect of pathogenic dystrophin variants on female carriers’ cognition and behavior, including ASD.

In summary, despite the challenges to diagnose ASD, its prevalence is clearly increased among dystrophinopathies. The higher prevalence and the spectrum of severity of ASD reinforce the relevance of dystrophin during neurodevelopment and indicate that pathogenic variants in the dystrophin gene render the brain more susceptible to ASD or even other NDDs or neuropsychiatry phenotypes commonly observed in DMD/BMD patients.

## Genotype–phenotype correlation

Several authors have tried to explain the NDD phenotype variability observed among DMD/BMDs, and one of the most common strategies was the analysis of dystrophin mutations’ site and its possible correlation with the NDD outcome. Although early studies did not find correlation between the longest and shorter isoforms [97, 98], it is now broadly accepted that pathogenic variants in the C-terminal portion of the dystrophin gene, affecting an increased number of isoforms, including the Dp140 and Dp71, in addition to the Dp427 isoforms, is associated with a higher prevalence and severity of intellectual disability among DMD/BMD individuals [71, 99]. However, individuals carrying the same dystrophin variant can present different cognitive/NDD outcomes [34, 97]. In regard to ASD, genotype–phenotype analysis were performed only by eight groups [34, 41, 47–49, 80, 82, 86], and 22.2% (2/9) of the analysis pointed for a positive correlation of ASD with Dp140 and/or Dp71 isoforms [34, 82], but access of these results was limited (Table 1). Although still modest and controversial, the current data on this topic suggest that other factors may contribute to the ASD phenotype in DMD/BMD patients.

## Description of new cases of ASD individuals harboring rare variants in the dystrophin gene

Here we describe four families (including six affected individuals) for which dystrophin rare LoF variants were found in ASD probands (clinical data summarized in Table 2). Dystrophin gene variants in two of these families (Families F1 and F2) were identified through the investigation of rare variants (both point mutations and CNVs) of whole exome sequencing (WES) performed in a cohort of 328 (65 females and 263 males) ASD Brazilian individuals (ASD genomic data in [11, 100, 101]). The two dystrophin variants identified in these two families were, respectively classified as pathogenic and of uncertain significance (VUS), following the American College of Medical Genetics (ACMG) guidelines [102]: an out-of-frame deletion encompassing exons 2–9 in a currently 17 year old male (P1; F1) and a stop gain variant in exon 78 in a currently 9 year old male (P3; F2) (Table 2). The deletion 2–9 is predicted to disrupt only the long Dp427 isoforms, while the variant in exon 78 may impair the majority of dystrophin isoforms. In this cohort, the frequency of variants in dystrophin is significantly increased (2/328; 0.61%) as compared to the frequency of DMD in the general population (~ 1:7000–10,000), a similar finding previously observed by Pinto et al. [30]. These data thus confirm the relevance of ASD in dystrophinopathies and the need to systematically analyze dystrophin variants in ASD cohorts as this approach can reveal relevant variants with a higher impact on the CNS than on muscle, as we will discuss below.

**Table 2:** Summarized clinical data of individuals from Family 1 (P1, P2), Family 2 (P3), Family 3 (P4, P5, P6) and Family 4 (P7, P8) reported in this review

Individual	Family 1		Family 2	Family 3			Family 4	
	P1	P2	P3	P4	P5	P6	P7	P8
Birth	9/12/2004	9/12/2004	8/5/2012	7/4/2009	7/4/2009	7/4/2009	13/06/2002	13/06/2002
Age at last evaluation	17 yo	17 yo	5 yo	8 yo	8 yo	8 yo	15 yo	15 yo
Weight	51 kg	62 kg	19.3 kg	17 kg	23.5 kg	23.5 kg	39 kg	32 kg
Stature	165.5 cm	171.5 cm	119 cm	100 cm	127 cm	122 cm	135 cm	130 cm
Head circumference	57 cm	57 cm	53 cm	52 cm	54 cm	54 cm	53 cm	NA
Wheelchair	No	No	No	No	No	No	9 yo	9 yo
Conception	Natural conception, no intercurrences reported		Natural conception, no intercurrences reported	In vitro fertilization, no reported intercurrence			Natural conception, no reported intercurrence	
Parental age at conception	Mother = 25 yo; father = 27 yo. Unrelated parents		Mother = 25 yo; Father = 26 yo. Unrelated parents	Mother = 40 yo; Father = 38 yo. Unrelated parents			Mother = 30 yo; Father = 43 yo. Unrelated parents	
Gestation	No intercurrences reported		No intercurrences reported	No intercurrences reported			No intercurrences reported	
Childbirth	Cesarean section (38th gestational week). P1 needed intensive care due to respiratory distress		Cesarean section (39th gestational week). Intensive care for 5 days due neonatal hypotonia and hypoglycemia	Cesarean section (34th gestational week) and hospital care for a month for weight gain			Natural section (37th), no intercurrences reported	
Head circunference	NA	NA	34 cm (p = 25th)	29.5 cm (p > 10th)	29 cm (p = 10th)	31 cm (50th > p > 10th)	NA	NA
Weight	2425 g (p < 10th)	2125 g (p < 5th)	2825 g (p > 25th < 50th)	1625 g (p = 3rd)	1745 g (p < 10th)	1920 g (p = 10th)	2330 g (p < 10th)	2350 g (p < 10th)
Stature	46 cm (p = 3th)	44 cm (p < 3th)	47 cm (p < 15th)	41 cm (p < 3th)	43 cm (p < 3th)	44 cm (p < 3th)	NA	NA
First words	13 months	13 months	9 months	Nonverbal	Nonverbal	Nonverbal	12 months	12 months
Walking independently	14 months	13 months	16 months	18 months	18 months	15 months	18 months	18 months
CK measure	NA	NA	NA	6775 U/L (year: 2012; ref: 26–189 U/L)	17,250 U/L (year: 2012; ref: 26–189 U/L)	56 U/L (year: 2012; ref: 26–189 U/L)	NA	24,850 U/L (year: 2010; ref: 26–189 U/L)
Clinical exams	Both auditory and EEG exams showed normal results	EEG, MRI, auditory and ophthalmologic exams all presented normal results	EEG, auditory, karyotype and Fragile-X exams were all normal. MRI showed the presence of arachnoid cyst (age = four yo)	NA	NA	NA	Myopia, cataract, vitamin D Deficiency, dyslipidemia, sinus tachycardia, presumed osteoporosis revealed by routine exams due their DMD diagnosis (age = 17yo). RMI and EEG not performed	Myopia, cataract, vitamin D Deficiency, dyslipidemia, sinus tachycardia, presumed osteoporosis revealed by routine exams due their DMD diagnosis (age = 17yo). RMI and EEG not performed
ASD diagnosis (neurologist, DMS-IV ou -5)	–	7 yo	3 yo	3 yo	3 yo	3 yo	4.5 yo	4.5 yo
CARS	26.5 (2019; no ASD)	35 (2019)	NA	33.5 (2016)	31 (2016)	32.5 (2016)	39 (2020)	39 (2020)
Other NDDs' diagnosis	intellectual disability interrogated	Intellectual disability interrogated	ADHD interrogated	Intellectual disability	Intellectual disability	Intellectual disability	–	–

*NA* information not available, *–* absent phenotype, *yo* years old; *ASD* autism spectrum disorder, *CARS* childhood autism rating scale

The two other ASD families, identified in our neuromuscular cohort, called our attention due the presence of a severe form of ASD among the patients diagnosed concomitantly or before DMD: one family, F3 composed by non-identical triplets, where the three boys are diagnosed with ASD (P4, P5, P6) and two of them have DMD (P4 and P5); the second case was ascertained as a pair of ASD-DMD monozygotic twins, family F4, (P7 and P8). The main clinical findings are summarized below and in Table 2.

### Family 1, P1 and P2 (discordant monozygotic twins)

Patient P1 was referred to our center at the age of 15 because of ASD. Interestingly, his co-monozygotic twin (P2) does not have ASD (Table 2). Despite being monozygotic twins, in addition to being discordant for the ASD phenotype, they currently have different anthropometric measurements (Table 2). P1 presented normal motor and speech development until 2 years and 6 months of age, when he started presenting speech, motor and behavioral regression, with evident echolalia. Tiptoe walking, common in both ASD and DMD, was also reported by the parents. He started to pronounce two words together again at 4 years of age, following professional help. A neurologist established ASD diagnosis when he was 7 years old. He attended a special school and currently he can read and write. ADOS-2 (23 points) and CARS (35 points) results were compatible with a mild to moderate ASD diagnosis. On the other hand, the co-twin P2 presented an unremarkable development. He attended regular school, although he always presented learning difficulties. CARS evaluation did not suggest autism (26.5). WES analysis, except for 2–9 dystrophin deletion, did not reveal any additional pathogenic ASD variant of major effect that could explain the ASD phenotype in P1. However, some potential disease risk variants were found including a potentially pathogenic variant for autosomal dominant late-onset dementia in *CSF1R* (#MIM 22180; Table 3). The 2–9 dystrophin deletion, initially detected through CNV analysis (NextGene® software/Softgenetics, USA) in WES’ P1, was validated by MLPA (multiplex ligation-dependent probe amplification) in both twin siblings. No other similar cases were reported in the family, but genetic testing revealed that the mother is a carrier of the dystrophin pathogenic variant. Given their unexplained and remarkable clinical differences, we expect that future studies will help to determine their dystrophin muscle and brain expression levels, in addition to the extension of this variant’s contribution to their phenotype. Moreover, the study of other mechanisms, such as epigenetic ones, may shed some light in understanding their different clinical outcomes.

**Table 3:** Rare potentially damaging or damaging exonic variants in P1 to P8 patients

Individuals	Gene	Chr	Start position	End position	Accession	Variant	§ gnomAD metrics	§ gnomAD metrics	1000 g frequency	gnomAD frequency	ABraOM frquency	CADD phred	Parental Origin	SFARI score	OMIM	ACMG classification	Clinvar
P1 and P2	*CSF1R*	chr5	150069942	150069942	NM_001288705	c.C1441T: p.Q481*	pLI = 0.13	Z-score = 1.57	0	0	8.21E–4	–	Paternal	–	Brain abnormalities, neurodegeneration, and dysosteosclerosis, 618476, AR; Leukoencephalopathy, diffuse hereditary, with spheroids, 221820, AD	Pathogenic (PVS1, PM2, PP5)	Pathogenic—rs917027829
	*WDFY4*	chr10	48790912	48790912	NM_001370153	c.T4252A:p.Y1418N	pLI = 0.34	Z-score = 3.15	0	0	0	29.1	Maternal	2	–	–	–
P3	*SQSTM1*	chr5	179836434	179836434	NM_001142298	c.914-2A > G	pLI = 0	Z-score = -0.94	0	0	0	34	Paternal	–	Frontotemporal dementia and/or amyotrophic lateral sclerosis 3, 616437, AD; Neurodegeneration with ataxia, dystonia, and gaze palsy, childhood-onset, 617145, AR; Myopathy, distal, with rimmed vacuoles, 617158, AR; Paget disease of bone 3, 167250, AD	Pathogenic (PVS1, PM2, PP3)	–
P4	*SPEN*	chr1	15928591	15928591	NM_015001	c.G2351A:p.R784H	pLI = 1	Z-score = 2.9	0.000599	0.0002	0.002	22.9	Maternal	2	Radio-Tartaglia syndrome, 619312, AD	Benign (BS1, BS2)	–
P5	*ATP2B2*	chr3	10350549	10350549	NM_001330611	c.G2030A:p.R677Q	pLI = 1	Z-score = 4.55	0.0002	0.0002	8.21E–4	23.2	Paternal	2	{Deafness, autosomal recessive 12, modifier of}, 601386, AR	Likely benign (BS1)	–
P5	*CHD3*	chr17	7899173	7899173	NM_001005271	c.A2491G:p.I831V	pLI = 1	Z-score = 6.15	0	0	0	23.2	Maternal	1	Snijders Blok-Campeau syndrome, 618,205, AD	Uncertain significance (PM1, PM2, PP2, PP3)	–
P5	*DLGAP1*	chr18	3879164	3879164	NM_001242761	c.C905T: p.A302V	pLI = 0.99	Z-score = 6.15	0.0002	0.0006	0	24	Paternal	2	–	–	–
P6	*SV2A*	chr1	149913420	149913420	NM_001328674	c.C421T: p.R141*	pLI = 0.2	Z-score = 2.12	0	4.07E-06	0	35	De novo	–	–	–	–
P6	*KDM4C*	chr9	7165299	7165299	NM_001304340	c.C2078G:p.P693R	pLI = 0	Z-score = − 0.49	0	0	0	25.8	Paternal	2	–	–	–
P4 and P6	*IRF2BPL*	chr14	77027039	77027039	NM_024496	c.G754A: p.V252M	pLI = 0.84	Z-score = 0.73	0	7.10E-06	0	22.8	Paternal	1	Neurodevelopmental disorder with regression, abnormal movements, loss of speech, and seizures, 618088, AD	Uncertain significance (PM2, PP2)	–
P4 and P6	*NRXN1*	chr2	50346736	50346736	NM_001330091	c.C214T: p.P72S	pLI = 1	Z-score = 2.56	0	0	0	20.5	Maternal	1	Pitt-Hopkins-like syndrome 2, 614325, AR;{Schizophrenia, susceptibility to, 17}, 614332	Uncertain significance (PM2)	–
P4 and P6	*CNTN4*	chr3	3040205	3040205	NM_001206956	c.G1345T:p.G449C	pLI = 0	Z-score = 0.57	0	4.06E-06	0	29.6	Paternal	2	–	–	–
P5 and P6	*ZC3H4*	chr19	47071997	47071997	NM_015168	c.G1927A:p.D643N	pLI = 1	Z-score = 0.66	0	0	0	23	Maternal	2	–	–	–
Shared by the triplets	*RANBP2*	chr2	108765416	108765416	NM_006267	c.G4877A:p.C1626Y	pLI = 1	Z-score = − 0.78	0	0	0	24.5	Maternal	–	{Encephalopathy, acute, infection-induced, 3, susceptibility to}, 608033, AD	Uncertain significance (PP3)	-
Shared by the triplets	*KALRN*	chr3	124398753	124398753	NM_001024660	c.A2222G:p.E741G	pLI = 1	Z-score = 4.36	0	0	0	27	Paternal	–	–	–	–
P7^a^	*EBF3*	chr10	129877788	129877788	NM_001005463	c.C616T: p.R206*	pLI = 1	Z-score 3.61	0	0	0	41	De novo	1	Hypotonia, ataxia, and delayed development syndrome, 617330, AD	Pathogenic (PVS1, PP5, PM2, PS2, PP3)	Pathogenic—rs1057519522

All variants reported are in known ASD (SFARI scores 1 and 2 genes, https://gene.sfari.org/database/human-gene/) and/or other NDDs genes. Variants’ selection was based on frequency (< 0.1%), type (loss of function or missense), and prediction of damaging in at least three programs and CADD > 20. pLI and Z-score were also used for their clinical interpretation. All variants met minimum quality criteria of > at least 20 reads, allele balance between 30 and 70%, genotype quality > 70, and GATK filter = PASS. ACMG classification was used in the context of OMIM associated diseases in order to determine the diseases' status. Human genome of reference = hg38. The dystrophin pathogenic variants in patients P1–P5, P7 and P8 are only described in the text

^a^WES analysis performed only for patient P7

### Family 2, individual P3

P3 was referred to our center for genetic counseling due to an ASD diagnosis when he was five years old. He was born from unrelated, healthy parents. Speech and motor development were unremarkable. However, at three years of age he presented stereotypes, echolalia, no interaction at school, and absent eye contact. His mother reported that he learned to read at three years, before starting school. After evaluation by a neurologist, he was diagnosed with high functioning ASD. No seizures, hypotonia or any clinical signs suggestive of muscular dystrophy were observed at the age of five years, when evaluated by us. WES revealed the above mentioned stop gain variant in dystrophin, NM_000109:c.11017A > T (p.Arg3673*), inherited from his mother. This variant is absent in males in the 1000Genomes, AbrAOM and GnomAD databases (variants of low quality were excluded), but it has been previously described in ClinVar (rs1477369230) as VUS in one DMD individual. This variant is predicted to create a premature stop codon at amino acid position 3673 (NM_000109) and may affect all the Dp427 transcripts as well as the shorter isoforms Dp140 and Dp71, known to be highly expressed in the brain. Since it is located at the 3′end of the dystrophin gene, it is difficult to predict its effect on the mRNA decay as well as its effect on each isoform. In addition, as recently shown by García-Rodriguez et al. [103], other mechanisms, such as epigenetic control, may regulate the expression of truncating dystrophin variants. Therefore, there is not yet enough information to understand its clinical impact on his neurodevelopmental phenotype. Of note, although WES analysis did not reveal any other variant that could explain his current phenotype, a pathogenic variant in a gene associated with autosomal dominant late onset dementia (*SQSTM1;* #MIM616437) was found (Table 3).

### Family 3, P4, P5 and P6: triplets

These three non-identical twin boys were conceived by in vitro fertilization (IVF) from healthy and non-consanguineous parents. None of the three brothers had developed verbal language until three years-old nor any nonverbal language to interact. They also typically displayed repetitive and stereotyped behavior. By the age of 3, all three brothers were clinically evaluated due to neurodevelopmental delay, in addition to the DMD hypothesis for two of them, who started walking at 18 months of age with frequent falls (brothers P4 and P5). At the age of three, they had global hypotonia, pseudohypertrophy of the calf muscles, flat feet and hyperlordosis. The third twin (P6) walked at 15 months of age. Creatine-kinase (CK) serum levels were in the normal range, and DMD diagnosis was excluded.

High serum CK levels (about 35–40 times increased) and muscle biopsy (dystrophic pattern with dystrophin deficiency, data not shown) in P4 and P5 were compatible with dystrophinopathy, which was confirmed via molecular testing, NM_000109: c.64_69 + 16delTCTAAGGTAAGAATGGTTTGTT (p.lys22_Ser23del). This variant possibly disrupts all the full-length isoforms, but not the shorter isoforms (Fig. 1). The two DMD boys, P4 and P5, at the age of six were scored 32.5 and 31, respectively by the CARS autism scale, which is compatible with ASD diagnosis. Vineland scores were 44 and 45, respectively, indicating low adaptive behavior. The non-DMD brother (P6) also fulfilled the ASD criteria with low adaptive behavior (CARS score = 32.5; and Vineland score = 50).

Since all siblings were diagnosed with ASD, but one of them does not have DMD, the pathogenic variant in this gene would not solely explain their neurodevelopmental phenotype. Array-CNV analysis did not reveal any pathogenic CNV of large clinical effect. WES analysis including their parents revealed no other pathogenic or potentially pathogenic variant that could explain their ASD phenotype, except for a de novo variant in *SVA2* in the non-DMD boy (P6). This gene encodes a membrane glycoprotein associated with synaptic vesicle trafficking and excitability [104–106], both molecular functions that once deregulated may predispose to ASD [107, 108]. Notably, a combination of different VUS were found in each one of the siblings (Table 3), suggesting that, in this case, a combination of genetic hits would be contributing to their phenotype as it would be expected for most of the ASD cases. We cannot, however, rule out epigenetic or genetic mechanisms undetectable by exome and array-CGH analysis.

### Family 4, P7 and P8: monozygotic twins

These two monozygotic DMD brothers were first referred to our center for genetic counseling when they were 8 years old. Both siblings presented delayed motor and speech development, with low anthropometric values (from 7 to 36 months, below the 3rd percentile). They started walking at 18 months of age, with reported repeated falls and remarkable imbalance. They both spoke their first words at around 12 months of age, with constant difficulties (slurred speech, echolalia), which required speech therapy.

In addition to motor and speech impairments, they also presented behavior and social problems, being both diagnosed with ASD when they were 4 years and 6 months old by a neurologist. They attended regular school only for one year and learned to read when they were three years old with their father's help. Although they always had difficulties in maintaining a dialogue, they can easily memorize songs and sing them, with the ability to memorize phrases. CARS evaluation for both twins showed results compatible with ASD diagnosis (CARS = 39 for both, performed when they were 17 years old). DMD diagnosis was made when they were eight years old, after clinical examination, elevated serum CK values and muscle biopsy. Molecular tests revealed the dystrophin stop gain variant: NM_000109:c.1591C > T (p.Arg531*), compatible with a molecular diagnosis of DMD. This variant is predicted to disrupt all the Dp427 dystrophin isoforms, but not the shorter ones. They stopped walking when they were nine years old. Clinically, they are remarkably similar.

WES analysis of one of the twins and their parents in the context of their neurodevelopmental disorders diagnosis revealed a heterozygous LoF pathogenic variant following ACMG criteria in *EBF3:* NM_001005463:c.616C > T (p.R206*). Heterozygous LoF variants in *EBF3* are associated with Hypotonia, ataxia, and delayed development syndrome (#MIM617330), and speech delay, hypotonia, gait or truncal ataxia, behavioral problems, including ASD are part of the phenotype. Multiple groups have first reported this syndrome in 2017 and currently only 42 individuals (from 39 families) have been described in the literature [109–115], in addition to the present family described here. More recently, non-coding variants affecting *EBF3* function were found in an ASD cohort [116] and two individuals carrying *EBF3* pathogenic LoF variants not associated with ID, but presenting autistic features [117] were described, expanding both the genetic and phenotypic landscape of ASD.

In short, we observed that only one of the six patients (P3) has a dystrophin variant that is predicted to disrupt all dystrophin isoforms, while in the other DMD patients (P1, P4–P7) the dystrophin variants are predicted to disrupt only the Dp427 isoforms. Despite the known relevance of the short isoforms (especially the brain expressed Dp140 and Dp71) to neurodevelopment, this observation suggests that all brain isoforms may contribute to ASD, including the longest ones.

## The genetic background to ASD in DMD patients

Our preliminary study reveals different scenarios regarding the genomic findings in these four families. In one of them (family 4; monozygotic twins P7 and P8)**,** two pathogenic variants of large effect were observed respectively in the dystrophin and *EBF3* genes. Notably, we did not raise the hypothesis that the phenotype of these twins were due to two independent pathogenic variants, possibly due to the high overlap of the clinical features observed in DMD and *EBF3* conditions. It is possible that the more severe ASD phenotype as in these twins is due to co-occurrence of different monogenic conditions, since the severity of ASD in these twins are not commonly seen among DMDs [41], as previously discussed. Another scenario observed was through the genomic analysis of the non-identical ASD triplets, which did not reveal any major pathogenic variant contributing to their neurodevelopmental phenotypes. Instead, different combinations of genetic risk ASD variants found in each individual supports an oligogenic/polygenic model underlying the psychiatric phenotype. Of note, these non-identical triplets also present a neuropsychiatric phenotype not commonly seen among DMD individuals, regarding severity.

Conversely, the other two families with no evident muscle phenotype revealed unusual rare dystrophin variants. The out-of-frame deletion encompassing exons 2–9 of the dystrophin gene identified in the proband of F1 has been described in only one asymptomatic female mosaic carrier [118] and not found in different databases [57, 119–121]. Dystrophin’s N-terminus deletions encompassing exons 3–9, which is the most similar variant as the 2–9 exon deletion here reported, have been associated with a great spectrum of clinical variability in DMD progression and there is no data on their impact in the brain [122]. Of note, the out-of-frame duplication of exon 2 was also associated with clinical variability in half brothers [123]. Interestingly, the monozygotic twins here reported are discordant for ASD, suggesting different regulatory mechanisms in brain and muscle, which would be caused by different factors, such as epigenetic changes under different environmental stress. Finally, although it is not expected that their DMD variant represents the major factor associated with their brain phenotype, it is possible that this variant (as well as other rare loss of function/DMD variants of unclear functional impact) represents a contribution factor in a more complex model, still to be elucidated. By contrast, the stop gain in dystrophin exon 78 was identified in family 2, with only a single report in the literature. Even though the clinical impact of this variant is unclear, it is possible that it has a larger effect in the brain than in muscle. Indeed, the description of a rare variant in the first exon of Dp71 associated with a neurodevelopmental phenotype alone [124] reinforces the hypothesis that some functional dystrophin variants have a larger effect in the CNS and a smaller or absent effect on muscle function. Interestingly, we identified two rare potentially pathogenic/pathogenic risk variants for dementia in these two cases, respectively in *CSF1R* and *SQSTM1*, which, although preliminary, call our attention to the possible relevance of genes associated with some types of late-onset neurological disorders not only for ASD [125, 126], but also for ASD-DMD/BMDs individuals as here described.

To date, very few studies have been performed taking into consideration the genetic background of DMD/BMD patients to reveal other factors potentially contributing to ASD or NDD phenotypes. Pagnamenta et al. [127] described two brothers diagnosed with ASD and without any muscle weakness at the time of evaluation who maternally inherited an in-frame duplication of exons 31–44 of the dystrophin gene, and paternally inherited an intragenic deletion of *TRPM3*, a potential ASD candidate gene [128]. Moreover, Karaca et al. [129] described two DMD brothers (with exons 46–47 deletions), both diagnosed with ID who, in addition to the dystrophin CNV, also presented a homozygous pathogenic missense variant in *DHCR7*, a known ASD gene also associated with Smith-Lemli-Opitz Syndrome (#MIM270400). Finally, Pizzo et al. [22] described a male, who carried a maternally inherited dystrophin LoF variant in addition to a de novo* SETD5* LoF variant, and a paternally inherited 16p12.1 deletion, being these last two known to represent variants of large and medium effect for ASD/NDD.

Although limited, the present study and the literature strongly suggest that the genetic background of DMD/BMD individuals may contribute to ASD, possibly with a heterogeneous and complex genetic architecture, as observed for ASD and other NDDs [22, 130, 131].

## Conclusions and future directions

ASD is a relevant endophenotype in dystrophinopathies, and its early diagnosis is important for a better management of the patient, with larger impact nowadays due increased longevity of the DMD patients. An increased prevalence of ASD among dystrophinopathies has been reported by several groups, but the heterogeneous estimates point to the need for well-established diagnostic guidelines, which will be particularly important for future genomic studies.

ASD genotype–phenotype studies with dystrophinopathies, although scarce, suggest the relevance of both longest and shorter dystrophin isoforms, differently to what has been observed for ID only. This is not unexpected, as the genetic architecture of ASD has its own signature, particularly in the non-monogenic forms [11]. Dystrophin isoforms and their regulation, which play important functional roles in neuronal and non-neuronal brain cells, certainly contribute to the ASD phenotype, but they may not be the only genetic factor.

Although limited, the genomic data in ASD-DMD patients suggest that the genetic background might contribute to the neuropsychiatric phenotype in these patients possibly under different models. Based on our data and in the literature, it is possible that severe ASD in DMD/BMD patients is caused by the combination of pathogenic variants of large effect in two independent *loci*, as exemplified by the *EBF3* LoF variant here reported in the DMD monozygotic twins. The co-occurrence of two or even three monogenic disorders has been reported by others with an estimated observed frequency of about 5–8.5% of the molecularly diagnosed cases by WES [25, 130]; however, as observed here and by others, identification of multiple molecular diagnoses can be missed due to clinical overlap [27, 132, 133]. Therefore, DMD patients with severe ASD forms should also be molecularly investigated to exclude the co-occurrence of another monogenic disorder. Contribution of variants of medium or lower effect also seems to be a possibility, as exemplified by the several at-risk ASD candidate variants in the non-identical triplets and the *TRPM3*-CNV reported by Pagnamenta et al. [127]. Therefore, it is possible that in addition to the dystrophin variant, variants of large and medium effects in neurodevelopmental genes are most often observed in the DMD/BMD patients with more severe forms of ASD than in those patients without any NDD, following an oligogenic model [24], although no systematic studies have yet addressed this question among DMD/BMD patients.

The relevance of the dystrophin LoF variants to the ASD phenotype but with smaller effect in the muscle phenotype has been poorly addressed to date. Enrichment of such dystrophin variants seems to be more likely observed in the ASD cohorts (this review and Ref. [127]). The identification and functional characterization of such variants in in vitro and in vivo models will be important to bring new insights into the function and regulation of dystrophin in the brain. Notably, the two ASD individuals with unusual dystrophin variants (P1 and P3) but no muscle phenotype at the original interview also harbor pathogenic variants in genes associated with late-onset dementia. Previous clinical and genetic studies have shown a higher incidence of dementia among adult ASD individuals, and different authors have addressed the genetic and molecular overlap between these two neurological conditions, although this relationship is still unclear [125, 126, 134]. In this context, a hypothetical explanation would be the interaction between a late-onset dementia pathogenic variant and an ASD/NDD gene variant, which should be investigated in larger cohorts.

The standardization of methods for the diagnosis of autism and genomic tools are fundamental to disentangle the heterogeneous and complex genetic architecture of ASD associated with dystrophin pathogenic variants, most particularly because dystrophinopathies are rare conditions and statistical power to uncover the genetic model underpinning ASD in these cases can only be achieved if consortia or meta-analyses are conducted. The relevance of early ASD diagnosis is undebatable, as early intervention contributes to a better quality of life of the autistic individuals. Finally, nowadays the possibility to model different variants in neuronal cells using CRISPR-Cas9 methodology together with neuronal cells differentiated from patients' induced pluripotent stem cells (iPSC) opens perspectives to test the genomic models, as well as to screen for molecules that could ameliorate the ASD phenotype.

## Data Availability

The datasets generated during and/or analyzed during the current study are available from the corresponding author on reasonable request.
